# Family planning needs to limit childbearing are unmet, yet our parity is high: characterizing and unveiling the predictive factors

**DOI:** 10.1186/s12905-023-02640-5

**Published:** 2023-09-15

**Authors:** A. S. Adebowale, M. E. Palamuleni

**Affiliations:** 1 Department of Epidemiology and Medical Statistics, Faculty of Public Health, University of Ibadan, Ibadan, Nigeria; 2 Population and Health Research Entity, Faculty of Humanities, North-West University, Mafikeng, South Africa

**Keywords:** Contraceptive use, High parity women, Unmet need to stop childbearing, Nigeria

## Abstract

**Background:**

The unmet need for limiting childbearing (UNLC) remains a problem in Nigeria. Conception after four pregnancies is considered a high-risk pregnancy. We examined the level, reasons for non-use of contraception, and predictors of UNLC among high parity (≥ 4 live birth) women in Nigeria.

**Methods:**

This cross-sectional design study was based on the analysis of nationally representative weighted data (2018 Nigeria Demographic Health Survey). The study focused on high-parity women of reproductive age (*n* = 4260) who do not want to have any more children irrespective of the number of their surviving children. Multi-stage cluster sampling approach was used for sample selection. Data were analyzed using logistic regression (α_0.05_).

**Results:**

Mean age of the respondents and children ever born was 38.92 ± 5.7 and 6.54 ± 2.3 respectively. The prevalence of UNLC was 40.9%, higher in the rural (48.8%) than urban (32.8%) areas, highest among women with no formal education (52.0%), higher among Muslims (48.4%) than Christians (34.8%), highest in the North-West (51.7%) and least in the South-East (26.1%). The most reported reasons for non-use of family planning (FP) were; respondents opposed (25.0%), infrequent sex (15.0%), fatalistic (13.2%), husband/partner opposed (11.2%), fear of side effects/health (8.5%), and religious prohibition (3.3%). The odds of UNLC was 100% higher among women aged 40–49 years compared to the younger women in age group 20–29 years. Living in the rural area predisposes high parity women of reproductive age to higher risks of UNLC (OR = 1.35, 95% C.I = 1.14–1.59, *p* < 0.001). Lack of access to family planning information through health workers (OR = 1.94, 95% C.I = 1.63–2.30, *p* < 0.001) increased the risks of UNLC. Being an Igbo or a Yoruba ethnic group was protective for UNLC compared to Fulani/Hausa women.

**Conclusions:**

A high level of UNLC was found among high-parity women in Nigeria. Access to FP information reduces the risk of UNLC. Expanding FP services would help respond to the expressed desires for contraception among high-parity Nigerian women who want to stop childbearing.

## Background

Unmet need for family planning (UNFP) is the proportion of women who are fecund, sexually active, and either want to limit or delay birth but are not using contraception. It measures the extent to which a country’s health system and cultural tendencies support women’s capacity to achieve their desired family size and quantifies the success of reproductive health programs in addressing the demand for services [1]. Its concept points to the difference between women's reproductive intentions and their contraceptive behavior [1]. UNFP is a global issue but its severity is more prominent in the low-income countries than the advanced countries. Across regions, UNFP ranges from 11% in the Middle East and North Africa to 26% in sub-Saharan Africa [2]. A recent study revealed a prevalence of 23.7% for UNFP in sub-Saharan Africa [3]. The UNFP in 12 Latin America and Caribbean countries varies from 6.1% in Peru to 30.5% in Guyana [4], 40.9% in Nepal, and 11.5% in Mexico [5, 6]. However, it was 9.6% in Botswana, 16.2% in Ethiopia, and 18.3% in Burkina Faso [7–9]. In Nigeria, UNFP was 18.0% but variation exists across sociocultural groups in the country [10] and it remains one of the reproductive health problems of international focus in contemporary times [11]. The adopted recommendations of the 1994 ICPD, the 1974 World Population Plan of Action, and the World Conference on Human Rights (1993) were clear about FP [12–14]. The FP-related issues subsumed in the themes of the Millennium Development Goals (MDGs) and Sustainable Development Goals (SDGs) may be unrealizable in Nigeria if issues that relate to UNFP are neglected [15, 16].

The Nigerian government has ratified some of these essential international treaties and adapted them into its national policies and guidelines [17–19]. This places an obligation on the government to provide high-quality contraceptive information, demand, and make services accessible to its enormous population. Nigeria has continued to exhibit its commitment to achieving the SDGs through leadership and ownership of the implementation process, however, the country continues to lag behind in some demographic indices that target the goals. The demographic dividend which is one of the hallmarks of this agenda might be delayed in a country like Nigeria where the contraceptive prevalence rate is low. Access to safe, voluntary FP is a human right because of its importance in gender equality, women’s empowerment, poverty reduction, and career advancement [20, 21]. Women who do not want to get pregnant either to space or limit childbearing at a time in their reproductive years have their peculiar reasons. However, it becomes a problem when such women are not using safe and effective FP methods, resulting in unwanted pregnancies. Research is consistent with the view that abortion is the major consequence of unintended pregnancy [22]. In many developing countries where there are laws that forbid abortion, pregnancy terminations are performed under unsafe conditions which often result in maternal mortality or lifetime disabilities [22, 23]. Where such pregnancies are not terminated, studies have identified delayed or no antenatal care, stigmatization, and family rejection among others as consequences. These practices can pose health risks and threats to the survival chances of both mother and infant [22, 24].

Nigeria with a TFR of 5.5 is among the high fertility countries worldwide, the population growth rate is high, and the contraceptive prevalence rate (15%) is abysmally low [10]. Nigeria is among the countries with the highest levels of UNFP worldwide despite being signatory to numerous FP international treaties. The high fertility and unintended pregnancies prevalence in Nigeria can be attributed to the low use of modern contraceptives. This poor reproductive health situation in the face of poverty which ravages the country often aggravates the risks of disability, morbidity, and mortality among women. Satisfying women’s unmet need for limiting childbearing (UNLC) requires identifying intra country populations where such need is high to facilitate programs and services that can effectively respond to the problem. Addressing their reasons for nonuse should inform FP programs’ efforts to fulfill the need. Studies have explored UNFP in Nigeria but they mainly focused on all women. Specific studies that target UNLC among high parity (≥ 4 live births) women are relatively scarce. Ideally, fecund, sexually active women who want to limit childbearing after attaining the stage of high-risk pregnancy should stop further conception. However, many such women could not achieve this desire on the account of non-use of modern contraceptives. It is thus imperative to know the characteristics, level, and predictors of UNLC among the women. The reasons for the non-use of contraception were also identified. Addressing these objectives will assist the policymakers to respond to the expressed fertility preferences of women. The program managers will find the outcome of this study valuable when evaluating and designing strategies for improving maternal health and slowing the population growth rate in Nigeria.

## Method

### Study area

This study was conducted in Nigeria, the African most populous country with a population figure of above 217 million and a population growth rate of 2.4% [25]. Women of reproductive age constituted (23%) of the Nigerian population [25]. The population is young consisting of a broad base pyramid with a narrow top. Nigeria is still regarded as a low-income country with poor demographic indices like maternal mortality ratio – 512 maternal deaths per 100,000 live births, infant mortality rate of 67 per 1,000 live birth, total fertility rate of 5.3 on average to a woman of reproductive age if the current age-specific fertility is sustained throughout the reproductive years, the median age at first sexual intercourse – 17.2 years, median age at first marriage – 19.1 years [10]. However, there is quite diversities in these indices at the regional level and across socioeconomic lines. The country has regions otherwise known as geo-political zones including North-Central, North-East, North-West, South-East, South-South, and South-West. The three main ethnic groups in the country are Hausa/Fulani, Igbo, and Yoruba and the people who belong to these ethnic groups are more dominant in the North East/North-West, South-East, and South-West respectively.

### Study design and population

This cross-sectional design study was based on the analysis of nationally representative data – 2018 Nigeria Demographic Health Survey, the most recent round of such survey in Nigeria. The survey was implemented by the National Population Commission (NPC) in collaboration with the National Malaria Elimination Programme (NMEP) of the Federal Ministry of Health, Nigeria [10]. The primary objective of the 2018 NDHS was to provide up-to-date estimates of basic demographic and health indicators – family planning inclusive. However, only women of reproductive age who had previously given birth to at least four children tagged as one of the indices of high-risk pregnancy were considered for analysis. In this study, the focus was on high-parity women (*n* = 4260) who do not want to have any more children irrespective of the number of their surviving children. The menopausal women, currently pregnant women, and those who are currently breastfeeding were excluded from the analysis because their inclusion can either bias or distort the outcome of this study.

### Data collection

Data collection took place from 14 August to 29 December 2018. The 2006 Population and Housing Census of the Federal Republic of Nigeria (NPHC) sampling frame as modified in inline with the recent demarcation structure was used. Administratively, Nigeria is divided into states. Each state is subdivided into local government areas (LGAs), and each LGA is divided into wards. During the 2006 NPHC, each locality was subdivided into convenient areas called census enumeration areas (EAs). The primary sampling unit (PSU), referred to as a cluster for the 2018 NDHS, is defined on the basis of EAs from the 2006 EA census frame. The sample used for the survey was a stratified sample selected in two stages. Stratification was achieved by separating each of the 36 states and the Federal Capital Territory into urban and rural areas. In total, 74 sampling strata were identified. Samples were selected independently in every stratum via a two-stage selection. In the first stage, 1,400 EAs were selected with probability proportional to EA size. In the second stage’s selection, a fixed number of 30 households was selected in every cluster through equal probability systematic sampling, resulting in a total sample size of approximately 42,000 households [10]. Further information on the sampling and data collection strategies for the survey is available for public consumption in the NDHS report [10].

### Variable definition

The dependent variable was UNLC. Reasons for the non-use of modern contraceptives among the study subjects were also explored. A woman has an unmet need for FP if she is fecund, sexually active, not using any contraceptive methods, and does not want a child for at least two years or wants no more children [1]. Thus, we focused mainly on the unmet need for limiting (stopping) childbearing. The independent variables included: Demographic: age, region, residence, marital status, marriage type, sex preference; Socioeconomic: level of education, ethnicity, wealth index, work status, household decision-making power, partner’s education, and religion. Media Related: media exposure, media access to family planning information, and access to family planning information from health workers.

The following variables were created using set of variables included in the data*.*

#### Sex preference

Sex preference was created as a proxy from variables on the ideal number of boys (v627) and ideal number of girls (v628). The ideal number of girls was subtracted from the ideal number of boys as v627 – v628. The result is either positive or zero or negative. Respondents with zero answers are considered as having no sex preference, while those with positive and negative results are considered to have a preference for boys and girls respectively. Thus the new variable called sex preference has three categories (None, females, males).

#### Household decision-making power

This was generated from the information on five items: a person who usually decides how to spend the respondent's earnings, the person who usually decides on the respondent's health care, the person who usually decides on large household purchases, the person who usually decides on visits to family or relatives, and the person who usually decides what to do with money husband earns. The response to each question has the following 5 options, husband/partner alone, respondent alone, respondent & husband/partner, respondent & another person, someone else. A score of 0 is assigned to the respondent if she picks either husband/partner or someone else. A score of 1 is assigned to either the respondent & husband/partner or the respondent & other person while 2 is assigned to the respondent when she is the only person who makes such a decision. Thus, the respondents’ score ‘x’ is a discrete variable 0≤x≤10. The overall score tagged as decision-making power was disaggregated into low, medium, and high for scores 0-4, 5-7, and 8-10 respectively.

#### Media exposure

The variable was generated based on the information provided by the respondents on the following 4 questions frequency of reading newspapers or magazines, frequency of listening to radio, frequency of watching television, and frequency of using the internet last month. The available options are: not at all=0, less than once a week=1, at least once a week=2, and almost every day=3. Thus, the maximum obtainable score ‘y’ is 12 and the least is 0 (0≤y≤12). The overall score tagged as media exposure was disaggregated into low, medium, and high for scores 0-5, 6-8, and 9-12 respectively.

#### Media access to family planning information

The variable was obtained from the combination of four variables. These are: heard family planning on the radio last few months, heard family planning on TV last few months, heard family planning in a newspaper/magazine in the last few months, and heard family planning by text messages on a mobile phone. The responses to each question are either ‘yes=1’ or ‘No=0’. Here, the maximum obtainable score is 4 but dichotomized as no access and access (at least one media).

#### Access to family planning information from the health worker

It was generated from the 4 variables including visits by the fieldworker in the last 12 months (v393), did fieldworker talk about family planning (v393A), visited the health facility last 12 months (v394), and the health facility, told of family planning (v395). Respondents who either did not visit health facilities or visited health facilities without any access to family planning information were assigned 0 score. Also those who were either not visited by the health worker or who were visited by the health worker without any discussion or talk about family planning were assigned a ‘0’ score. Those who had access to family planning information either on a visit to the health facility or during the visit of the health worker to their community were assigned a score of ‘1’. Consequently, the variable ‘access to family planning information from health worker’ was dichotomized as ‘0’ if no access and ‘1’ if otherwise.

### Data analysis

Due to the non-proportional allocation of the sample to different states in Nigeria and the possible differences in response rates, sampling weights were calculated so that the results would be representative at both the national and domain level. This is with the view to generate design-unbiased estimates of population parameters. Data were analyzed using descriptive statistics, Chi-square tests, and binary logistic regression (α = 0.05). Percentage frequency distribution was used to describe the characteristics of the studied women according to UNLC by background characteristics. The reasons for the non-use of family planning methods among those women who have a UNLC, the contraceptive methods currently used among those with the met need for limiting childbearing, and the fertility experience of all the subjects were also described with the use of frequency distribution and descriptive statistics where necessary. The Chi-square test was conducted to examine an association between UNLC and each of the independent variables.

The logistic regression model was used to identify the determinants of UNLC among the high-parity women in Nigeria. In this context, three models were constructed. The first is the bivariate logistic regression where the model was restricted to only two variables, the dependent and the independent variables. The second and third models are multivariate logistic regression where only media-related variables (media exposure, media access to family planning information, and access to family planning information from health worker) and all variables were included in the model respectively.

## Results

The distribution of high-parity women according to UNLC by demographic characteristics is presented in Table 1. The mean age of the respondents and children ever born was 38.92 ± 5.7 and 6.54 ± 2.3 respectively. The prevalence of UNLC was 40.9% and this increases consistently from 28.2% among women in the age group 20–29 years to 46.5% in the age group 40–49 years. The UNLC was higher in the rural (48.8%) than in urban (32.8%) (*p* < 0.001); highest in the North-West (51.7%) and least in the South-East (26.1%) among the six regions in Nigeria (*p* < 0.001). The proportion of women with an UNLC was slightly higher among those in polygamous (42.8%) marriage compared to their counterparts in monogamous (39.1%) marriage (*p* = 0.042).

**Table 1:** Percentage distribution of high parity women according to unmet needs for limiting childbearing by demographic characteristics

**Background**	**UNLC**	**Total Number of**	${{\varvec{\chi}}}^{2}-{\varvec{v}}{\varvec{a}}{\varvec{l}}{\varvec{u}}{\varvec{e}}$	***p*-value**
**Characteristics**	**Yes**	**women**		
**Total**	**40.9(1471)**	**4262**		
**Age**			61.594*	< 0.001
20–29	28.2(61)	216		
30–39	35.9(681)	1895		
40–49	46.5(999)	2148		
**Region**			141.55	< 0.001
North Central	42.9(279)	650		
North East	49.7(228)	459		
North West	51.7(491)	949		
South East	26.1(177)	678		
South South	42.2(236)	559		
South West	34.2(331)	967		
**Residence**			113.760	< 0.001
Urban	32.8(693)	2115		
Rural	48.8(1047)	2144		
**Marital Status**			206.277	< 0.001
Never in union	10.7(3)	28		
Currently Married	44.3(1714)	3871		
Formerly Married	6.4(23)	360		
**Marriage Type**			4.136	0.042
Monogamy	39.6(1037)	2616		
Polygamy	42.8(703)	1643		
**Sex Preference**			1.774	0.412
None	41.3(1213)	2935		
Female	41.5(200)	482		
Male	38.8(327)	842		

The distribution of high-parity women according to unmet needs for limiting childbearing by socioeconomic characteristics is presented in Table 2. The UNLC was highest among women with no formal education (52.0%) and lowest among those with higher education (26.4%). Among the ethnic groups, Fulani/Hausa (53.4%) has the highest while Yoruba has the least (33.8%) UNLC; and it was higher among Islam (48.4%) than Christians (33.0%). About 52.0% of the women who belong to poor household wealth have UNLC compared to 32.4% observed among those who are rich. UNLC was 44.3% and 29.3% among women who have no access and those who have access to FP information from a health worker in the past 12 months prior to the survey respectively.

**Table 2:** Percentage distribution of high parity women according to unmet needs for limiting childbearing by socioeconomic characteristics

**Background**	**UNLC**	**Total Number of**	${{\varvec{\chi}}}^{2}-{\varvec{v}}{\varvec{a}}{\varvec{l}}{\varvec{u}}{\varvec{e}}$	***p*-value**
**Characteristics**	**Yes**	**women**		
**Total**	**40.9(1471)**	**4262**		
**Level of Education**			136.732	< 0.001
No education	52.0(750)	1441		
Primary	40.2(449)	1118		
Secondary	33.1(457)	1379		
Higher	26.4(85)	322		
**Ethnicity**			168.128	< 0.001
Fulani/Hausa	53.4(615)	1151		
Igbo	26.8(228)	851		
Yoruba	33.8(305)	902		
Others	43.7(593)	1356		
**Media Exposure**			51.968	< 0.001
None	47.5(546)	1149		
Poor	39.8(1121)	2818		
Good	25.3(74)	293		
**Wealth Index**			130.726	< 0.001
Poor	52.0(692)	1330		
Middle	43.3(392)	905		
Rich	32.4(656)	2024		
**Media Access to FP information**			12.849	< 0.001
None	43.1(1084)	2514		
Access	37.6(657)	1746		
**Access to FP information from health worker**			71.120	< 0.001
None	44.3(1454)	3279		
Access	29.3(287)	981		
**Work status**			3.112	0.078
Not working	43.8(310)	707		
Working	40.3(1431)	3553		
**Household decision making power**			50.482	< 0.001
Low	48.7(1107)	2271		
Medium	35.9(392)	1091		
High	41.7(195)	468		
**Partner’s Education**			117.83	< 0.001
No education	57.5(565)	983		
Primary	44.9(351)	782		
Secondary	38.2(568)	1487		
Higher	34.7(192)	554		
Don't know	58.5(38)	65		
**Religion**			82.111	< 0.001
Christian	34.8(808)	2320		
Islam	48.4(928)	1919		
Others	23.8(5)	21		

**Fig. 1:**
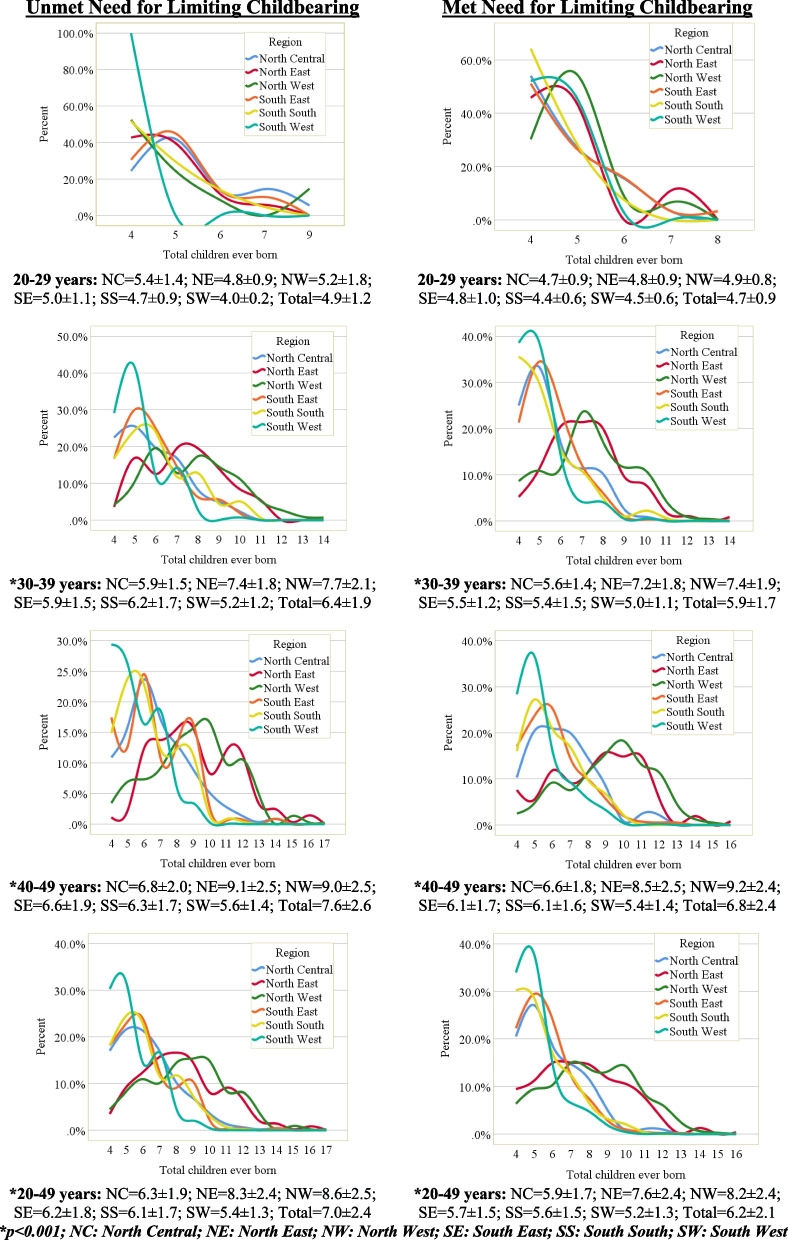
Percentage distribution of high parity women who have unmet and met need for limiting childbearing by children ever born according to regions in Nigeria

The standardized age pattern of children ever born and mean children ever born among high parity women aged 20–49 years who have UNLC and Met Need for Limiting Childbearing (MNLC) across the six regions in Nigeria is presented in Fig. 1. Among women in the age group, 20–29 years who have UNLC, the mean children ever born was highest in the North Central (5.4 ± 1.4) and lowest in the South West (4.0 ± 0.2 years). For those in the age group 30–39 years and 40–49 years, the mean children ever born was higher in all the regions in the North than the South. For the women in the age group 30–39 years, the highest mean children ever born was found among those in the North West (7.7 ± 2.1) and least in the South West (5.2 ± 1.2) region. A similar pattern was observed for the children ever born of women in the age group 40–49 years, while the North East women (9.1 ± 2.5) had the highest children ever born, the least children ever born was observed among their counterparts in the South West (5.6 ± 1.4). Combining all the age groups from 20 to 49 years, the mean children ever born in increasing magnitude was found to be 5.4 ± 1.3 years, 6.1 ± 1.7, 6.2 ± 1.8, 6.3 ± 1.9, 8.3 ± 2.4, and 8.6 ± 2.5 among women who reside in the South West, South South, South East, North Central, North East, and North West respectively. Across the age groups, the mean children ever born increases consistently from 4.9 ± 1.2 children among women in the age group 20–29 years to 7.6 ± 2.6 children among those in the age group 40–49 years.

The pattern of distribution of children ever born found among women who have UNLC was also observed among their counterparts with MNLC. However, across all age groups – 20–29, 30–39, and 40–49 years, there was a consistently higher mean number of children ever born among women who have UNLC than those who have MNLC. In the age group 20–29 years, 30–39 years, while the mean children ever born to women who have UNLC was 4.9 ± 1.2, 6.4 ± 1.9, and 7.6 ± 2.6, it was 4.7 ± 0.9, 5.9 ± 1.7, and 6.8 ± 2.4 children respectively among their counterparts who have MNLC. Overall, the mean children ever born among women aged 20–49 years who have UNLC (7.0 ± 2.4) was significantly higher than that of women who have MNLC (6.2 ± 2.1).

**Fig. 2:**
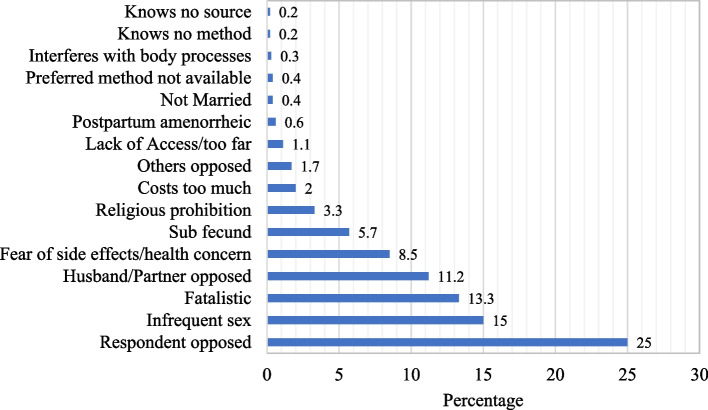
Percentage distribution of high parity women who are not using FP method according to the reasons for non-use (*n* = 1600)

Figure 2 shows the percentage distribution of high-parity women who are not using the FP method according to the reasons for non-use. The data depict that the mostly reported reasons for non-use of FP among those who have an UNLC were; respondent opposed (25.0%), infrequent sex (15.0%), fatalistic (13.3%), husband/partner opposed (11.2%), fear of side effect/health concerns (8.5%), religious prohibition (3.3%), costs too much (2.0%).

**Fig. 3:**
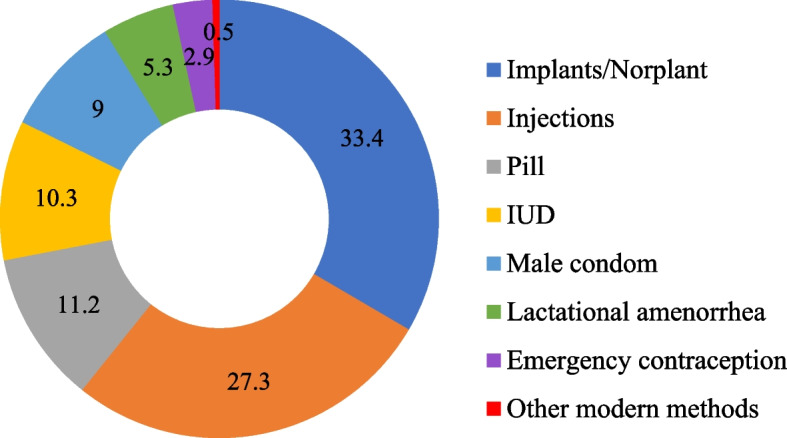
Percentage distribution of high parity women who are currently using modern contraceptive method according to type of method (*n* = 1123)

The distribution of high-parity women who are currently using the modern contraceptive methods according to the type of contraceptive used is presented in Fig. 3. Among the 1123 women who were currently using contraceptive methods, the data show that Implants/Norplant (33.4%), Injections (27.3%), Pills (11.2%), and IUDs (10.3%) are the highest four contraceptive methods currently being used. Others included Male condoms (9.0%), Lactational amenorrhea (5.3%), and Emergency contraception (2.9%).

**Fig. 4:**
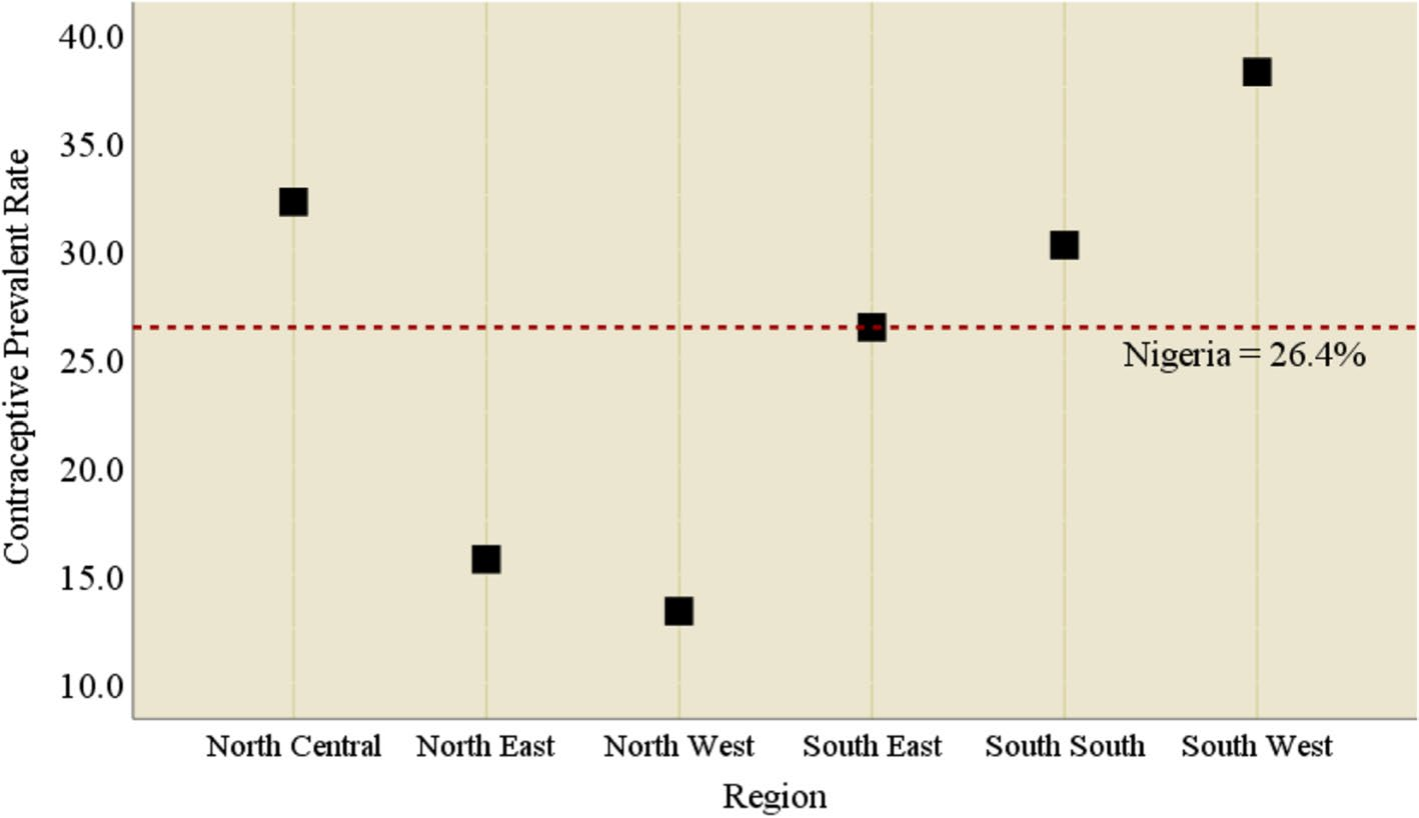
Contraceptive prevalence rate among high parity women across the six regions in Nigeria

At the national level, the prevalence of modern contraceptive use among the respondents was 26.4% and only the North East and North West had prevalence that was below this level. Women in the South West (38.2%) have the highest contraceptive use prevalence rate, their counterparts in the North West had the least (13.3%). The prevalence was found to be 32.2%, 30.2%, 26.4%, and 15.7% in the North Central, South South, South East, and North East respectively (Fig. 4).

In Table 3, the unadjusted and adjusted models of the determinants of UNLC were presented. Model 1 is the bivariate analysis to establish if a relationship exists between each background variable and UNLC, while Model 2 and Model 3 are multivariate outputs where only media-related variables and all variables were included in the model respectively.

**Table 3:** Logistic regression model of the factors influencing unmet need to limit childbearing among high parity women in Nigeria

Background	Model 1	Model 2	Model 3
**Characteristics**	**uO.R(95% C.I)**	**aO.R(95% C.I)**	**aO.R(95% C.I)**
**Age**			
20–29	1.00		1.00
30–39	1.42(1.04–1.94)***		1.31(0.96–1.88)
40–49	2.21(1.62–3.00)*		2.01(1.43–2.99)**
**Region**			
North Central	1.44(1.17–1.77)*		1.19(0.88–1.60)
North East	1.89(1.51–2.37)*		1.08(0.74–1.56)
North West	2.06(1.71–2.47)*		0.92(0.64–1.31)
South East	0.68(0.54–0.84)*		1.10(0.72–1.67)
South South	1.40(1.13–1.74)**		1.61(1.15–2.23)**
South West	1.00		1.00
**Residence**			
Urban	1.00		1.00
Rural	1.96(1.72–2.22)*		1.35(1.14–1.59)*
**Level of Education**			
No education	3.02(2.30–3.95)*		1.21(0.83–1.76)
Primary	1.87(1.41–2.46)*		1.30(0.92–1.84)
Secondary	1.38(1.05–1.81)***		1.33(0.96–1.84)
Higher	1.00		1.00
**Ethnicity**			
Fulani/Hausa	1.00		1.00
Igbo	0.32(0.26–0.39)*		0.54(0.34–0.84)**
Yoruba	0.45(0.37–0.53)*		0.66(0.45–0.94)***
Others	0.68(0.57–0.79)*		0.85(0.64–1.13)
**Media Exposure**			
None	2.68(2.01–3.58)*	2.50(1.84–3.39)*	0.95(0.65–1.38)
Poor	1.95(1.48–2.57)*	1.91(1.44–2.52)*	1.09(0.78–1.51)
Good	1.00	1.00	1.00
**Wealth Index**			
Poor	2.26(1.96–2.61)*		1.44(1.15–1.79)**
Middle	1.59(1.35–1.87)*		1.26(1.03–1.54)***
Rich	1.00		1.00
**Media Access to FP information**			
None	1.26(1.11–1.42)*	1.02(0.88–1.17)	0.91(0.77–1.06)
Access	1.00	1.00	1.00
**Access to FP information from health worker**			
None	1.93(1.65–2.25)*	1.86(1.59–2.17)*	1.94(1.63–2.30)*
Access	1.00	1.00	1.00
**Marital Status**			
Never in union	1.76(0.50–6.15)		1.56(0.30–5.13)
Currently Married	11.40(7.46–17.41)*		7.32(4.49–12.46)*
Formerly Married	1.00		1.00
**Marriage Type**			
Monogamy	1.00		1.00
Polygamy	1.14(1.01–1.29)***		1.27(1.08–1.49)**
**Sex Preference**			
None	1.11(0.94–1.30)		
Female	1.12(0.88–1.40)		
Male	1.00		
**Work status**			
Not working	1.16(0.98–1.36)		
Working	1.00		
**Household decision making power**			
Low	1.33(1.08–1.63)**		1.07(0.85–1.33)
Medium	0.79(0.62–0.98)***		0.83(0.65–1.05)
High	1.00		1.00
**Partner’s Education**			
No education	1.00		1.00
Primary	0.60(0.49–0.73)*		0.99(0.78–1.25)
Secondary	0.46(0.38–0.54)*		0.83(0.66–1.03)
Higher	0.39(0.31–0.49)*		0.72(0.54–0.96)***
Don't know	1.04(0.62–1.74)		1.76(1.01–3.07)***
**Religion**			0.99(0.78–1.25)
Christian	1.00		1.00
Islam	1.75(1.54–1.98)*		1.17(0.94–1.46)
Others	0.59(0.21–1.65)		0.65(0.21–1.97)
***-2 Log likelihood***		***5643.580***	***4815.444***^***a***^
***Cox & Snell R***^***2***^		***0.027***	***0.109***
***Nagelkerke R***^***2***^		***0.037***	***0.146***

^*^p < *0.001*

^**^p < *0.01*

^***^p < *0.05*

In Model 3 which is considered the full model, the identified predictors of UNLC were; age, region, residence, ethnicity, wealth index, access to FP information from health workers, marital status, family type, and partner’s/husband’s education. The odds of UNLC was 2.01(95% C.I = 1.43–2.99, *p* < 0.01) times higher among women aged 40–49 years compared to the younger women in the age group 20–29 years. Living in a rural area predisposes women to higher risks of UNLC (OR = 1.35, 95% C.I = 1.14–1.59, *p* < 0.001). However, being an Igbo (OR = 0.54, 95% C.I = 0.34–0.84, *p* < 0.01) or a Yoruba (OR = 0.66, 95% C.I = 0.45–0.94, *p* < 0.05) ethnic group was protective for UNLC compared to Fulani/Hausa women. The likelihood of having UNLC falls consistently with increasing levels of household wealth as it was 44% (OR = 1.44, 95% C.I = 1.15–1.79, *p* < 0.01) and 26% (OR = 1.26, 95% C.I = 1.03–1.54, *p* < 0.05) higher among women in poor and middle household wealth respectively. A similar pattern was observed with partner/husband’s level of education. Lack of access to family planning information through health workers (OR = 1.94, 95% C.I = 1.63–2.30, *p* < 0.001) and being in a polygamous relationship (OR = 1.27, 95% C.I = 1.08–1.49, *p* < 0.01) increased the risks of UNLC (Table 3).

## Discussion

Women with UNLC are those who are fecund and sexually active but are not using any method of contraception and report not wanting any more children. This points to the gap between women's reproductive intentions and their contraceptive behavior. In Nigeria, the knowledge of family planning methods is high, but its uptake is abysmally low despite the efforts of government and international agencies aimed at increasing this fertility control mechanism [18, 19]. It is worrisome that some high-parity women who believed that they have attained their desired family size and are willing to use family planning are not currently using any methods. Consequently, we examined factors responsible for UNLC in Nigeria, a country with an accelerated population growth rate. We found that about two-fifth of the high-parity women in Nigeria have UNLC. However, variation existed across the six regions in Nigeria with the regions in the North particularly North-East and North-West having a higher prevalence of UNLC than the South. This finding corroborates the outcome of similar research conducted in Togo, a country in West Africa [26–28]. The sociocultural diversities in the Northern and Southern regions of Nigeria could be responsible for the difference. In this study, UNLC was also more prevalence among women who belong to the Islamic religious groups which mostly dominate the Northern regions than those who are Christians which is a more common practice among the Southerners.

In terms of access to healthcare and health care related information, rural dwellers are consistently inhibited than the urban population, possibly because of fewer service providers, higher illiteracy levels, and less income in the rural areas [10, 29]. Research is persistent with the view that people who live in rural areas are more likely to engage in risky health-seeking behaviors including childbearing [30]. In the current study, the data showed that the UNLC was higher in the rural than in the urban area. These findings perfectly support the outcome of previous studies conducted in Nigeria [31] and elsewhere [29]. Differences in access and use of health services may be attributed to the socioeconomic disparities between rural and urban areas. In addition, structural barriers such as a shortage of skilled healthcare providers and limited media exposure may make it challenging for rural residents to access health information, especially those with limited health literacy.

The UNLC increases consistently with an increasing level of education. Slightly above half of women with no formal education are having UNLC compared to a quarter observed among their counterparts with tertiary education. Higher education increases the proximity to health information access and the ability to diagnose social and health consequences of either social or health risk behaviors including high fertility [32, 33]. The need to adopt family planning methods to stop childbearing because of career development may be much more necessary among the more educated women with high parity than their less educated counterparts. In Nigeria, more educated women are engaged in white-collar jobs that may not provide an avenue for frequent childbearing and childrearing. Therefore, in this context, family planning becomes a necessity in order to retain their job. A similar pattern to women’s education regarding the UNLC was observed for the partner’s/husband’s education. Nigeria is a patrilineal society where most household decisions are made by men [34, 35]. Thus, there is no doubt that highly educated men are better informed and have access to quality health education, particularly on the need to maintain a small family size than poorly educated men. Clear knowledge and understanding of socioeconomic and health consequences of large family size among the more educated men [34] may have a positive influence on family planning uptake either by themselves or their spouses.

Women of reproductive age recurrently come in contact with healthcare facilities especially to seek advice and they are also targeted for reproductive health-related issues by the community health workers. During such discussions, they are often privileged to be informed about FP and the consequences of high fertility. Access to such information can erode the cultural orientation and perspectives of women toward FP uptake. In the current study, we found that access to FP information from health workers at either the health facility or community level was protective against UNLC among high-parity women in Nigeria. Higher access and use of FP among those who have received FP information through health workers have been reported in earlier research [29, 31, 36]. However, the prevalence of UNLC found in our study was lower than what was obtained in the previous studies and this difference can be attributed to the subjects of focus. While our study focuses on high parity women of reproductive age, other studies based their analysis on the entire spectrum of women of childbearing age [29, 31, 36].

Aside from place of residence, education, partner’s/husband’s education, and access to family planning information from health workers, other predictors of UNLC found in this study included age, ethnicity, wealth index, marital status, and family type. The older age group (40–49 years) was more predisposed to the risk of UNLC than the younger age group (20–29 years). Hausa/Fulani women were more likely to experience UNLC than their counterparts who belong to Yoruba and Igbo ethnic groups. Cultural differences and discrepancies in the literacy level between Hausa/Fulani and Yoruba/Igbo women [10] are possible factors for the disparity in the risk of UNLC among these ethnic groups. The likelihood of UNLC was inversely related to household wealth with reducing UNLC as the level of household wealth increases. Family planning demand is not totally free in terms of cost in Nigeria. The costs that are associated with the transportation to the health facility and also procurement of certain contraceptive methods like pills, condoms, injectibles, etc. can limit poor women's access to family planning uptake [37]. Consequently, high parity women from richer household wealth might be at an advantage in contraceptive access and use over their counterparts who are poor. This study also revealed that being in a monogamous family was more protective against UNLC than polygamy. A social review of family issues as pointed out by Adebowale and colleagues showed that childbearing and gender preference are more prominent in polygamous families due to competition among the wives [38]. High-parity women from such families may decide not to use contraceptive methods even when they have the intention to limit childbearing. The direction of our findings with respect to all these factors have been consistently reported in the literature [3].

This study further revealed that the fertility experience of high parity women as measured by children ever born was strikingly higher in the North-East and North-West than regions in the South. The regional difference in fertility has been established in studies conducted in other countries in Africa [39] and our finding perfectly aligns with the results of a national study previously conducted in Nigeria. This outcome is expected since the prevalence rate of modern contraceptives was least and UNLC was the highest in these two Northern regions in Nigeria. Earlier studies have reported a higher proportion of women who had experienced early marriage, early sexual intercourse, and early childbearing in these two regions than in any other region in Nigeria [40, 41]. In such circumstances, longer duration of exposure to the risk of childbearing and short birth interval are implicated as reasons for higher fertility [42]. In addition, the women in the North East and North West are predominantly Muslim which are characterized by low use of modern contraceptives and early marriage [10].

At the national level, approximately one-fourth of the high parity women being investigated were currently using modern contraceptive methods and only the North East and North West had a prevalence that was below this level. However, modern contraceptive use was highest in the South-West. These findings are consistent with what literature had documented with respect to the regional pattern of contraceptive use in Nigeria [43]. The most cited family planning methods currently being used by the study subjects were implants/norplant, injections, pills, IUDs, male condoms, lactational amenorrhea, and emergency contraception in reducing order of magnitude. It is not surprising that high-parity women are mostly using long-acting contraceptive methods like implants/norplant, injections, and IUDs. It is therefore convincing that the women investigated in this study are doing this to stop childbearing since they do not have a plan to bear more children. However, among those who are not currently using any family planning method, the mostly reported reasons for non-use were; respondent opposed, infrequent sex, fatalistic, husband/partner opposed, fear of side effects/health concerns, religious prohibition, and the issues related to cost. It is amazing and frightening that high parity women who already signified that they would like to halt childbearing still provided these reasons for their non-use of contraception, despite numerous campaigns and programs on family planning uptake and availability of method that inimitably aligns with individual’s biological necessities in Nigeria [18, 19]. The identified barriers to the use of family planning found in the current study have been consistently reported in the literature [44–46].

### The policy implication of the findings

The high UNLC among high parity women of reproductive age found in this study, particularly in the Northern regions emphasizes the need to do more than just supply contraceptive commodities. Regional-specific issues around barriers to FP and contraceptive choices must be adequately addressed. It is also important to factor into the FP the identified reasons why high-parity women are not using contraceptives. Interventions that can facilitate improvement in FP service delivery, quality, and use may address UNLC in the short term. However, these activities must be accompanied by change in behavior and demand creation while meeting the supply needs.

### Limitations

The quantitative approach used in the current study may hide some contextual facts regarding the reasons for the non-use of FP among the investigated high-parity women. Therefore, a well-designed qualitative study is strongly recommended to fill this important gap. It is pertinent to note that the high level of UNLC found in this study should not be interpreted as the failure of the FP programs in Nigeria due to its flexibility. This is because the decline in fertility preference is yet to be achieved as a result of the high demand for large family size in Nigeria.

## Conclusion

A high level of UNLC was found among high parity women in Nigeria but was mostly prominent in the core Northern regions and among women who belong to Islamic religious denominations. Access to FP information either on media or through health workers reduces the risk of UNLC. Some high-parity women in Nigeria have a fatalistic attitude towards FP and there was an indication that women are obliged to non-use of FP because of self-opposition to use. Expanding and improving FP services would help to respond to the expressed desires for contraception among high-parity Nigerian women who want to stop having children. Addressing UNLC will improve family, maternal, and child health in the country. This will also provide couples with the means to limit the size of their families with the view to enhancing the overall socioeconomic development efforts in Nigeria. The characteristics of high parity women with an UNLC differ between Nigerian population subgroups. Therefore, it is pertinent that FP programmers and policymakers take a critical look at the peculiarity of this situation by developing suitable policies, strategies, and programs that will improve FP use.

## Data Availability

The datasets generated and/or analyzed during the current study are available in the DHS program Demographic and Health Survey repository, (https://dhsprogram.com/data/available-datasets.cfm).

## Abbreviation

UNLC: Unmet Need for Limiting Childbearing
FP: Family Planning
ICPD: International Conference on Population and Development
LGA: Local Government Area
